# Combined artificial high-silicate medium and LED illumination promote carotenoid accumulation in the marine diatom *Phaeodactylum tricornutum*

**DOI:** 10.1186/s12934-019-1263-1

**Published:** 2019-12-02

**Authors:** Zhiqian Yi, Yixi Su, Paulina Cherek, David R. Nelson, Jianping Lin, Ottar Rolfsson, Hua Wu, Kourosh Salehi-Ashtiani, Sigurdur Brynjolfsson, Weiqi Fu

**Affiliations:** 1 Center for Systems Biology and Faculty of Industrial Engineering, Mechanical Engineering and Computer Science, School of Engineering and Natural Sciences, University of Iceland, Reykjavík, 101 Iceland; 2 Department of Orthopaedics, Tongji Hospital, Tongji Medical College, Huazhong University of Science and Technology, Wuhan, 430030 China; 3 Center for Genomics and Systems Biology and Division of Science and Math, New York University Abu Dhabi, 129188 Abu Dhabi, United Arab Emirates; 4 Key Laboratory of Biomass Chemical Engineering of the Ministry of Education, College of Chemical and Biological Engineering, Zhejiang University, Hangzhou, 310027 China; 5 Biomedical Center and Department of Anatomy, Faulty of Medicine, University of Iceland, Reykjavík, 101 Iceland

**Keywords:** Carotenoid, Diatoms, Silicate, Fucoxanthin, Morphology, LED light

## Abstract

**Background:**

Diatoms, which can accumulate large amounts of carotenoids, are a major group of microalgae and the dominant primary producer in marine environments. *Phaeodactylum tricornutum*, a model diatom species, acquires little silicon for its growth although silicon is known to contribute to gene regulation and play an important role in diatom intracellular metabolism. In this study, we explored the effects of artificial high-silicate medium (i.e. 3.0 mM sodium metasilicate) and LED illumination conditions on the growth rate and pigment accumulation in *P. tricornutum*, which is the only known species so far that can grow without silicate. It’s well known that light-emitting diodes (LEDs) as novel illuminants are emerging to be superior monochromatic light sources for algal cultivation with defined and efficient red and blue lights.

**Results:**

Firstly, we cultivated *P. tricornutum* in a synthetic medium supplemented with either 0.3 mM or 3.0 mM silicate. The morphology and size of diatom cells were examined: the proportion of the oval and triradiate cells decreased while the fusiform cells increased with more silicate addition in high-silicate medium; the average length of fusiform cells also slightly changed from 14.33 µm in 0.3 mM silicate medium to 12.20 µm in 3.0 mM silicate medium. Then we cultivated *P. tricornutum* under various intensities of red light in combination with the two different levels of silicate in the medium. Higher biomass productivity also achieved in 3.0 mM silicate medium than in 0.3 mM silicate medium under red LED light irradiation at 128 μmol/m^2^/s or higher light intensity. Increasing silicate reversed the down-regulation of fucoxanthin and chlorophyll *a* under high red-light illumination (i.e. 255 μmol/m^2^/s). When doubling the light intensity, fucoxanthin content decreased under red light but increased under combined red and blue (50:50) lights while chlorophyll *a* content reduced under both conditions. Fucoxanthin accumulation and biomass productivity increased with enhanced red and blue (50:50) lights.

**Conclusion:**

High-silicate medium and blue light increased biomass and fucoxanthin production in *P. tricornutum* under high light conditions and this strategy may be beneficial for large-scale production of fucoxanthin in diatoms.

## Background

Diatoms are unicellular microalgae, which provide approximately half of the marine primary food sources. To date, diatoms have drawn increasing attention due to their plentiful practical applications in food, pharmaceutical and material industries. Diatoms can be easily cultivated, and they usually accumulate high content of bioactive compounds, such as essential fatty acids and carotenoids [1, 2]. Fucoxanthin, as a xanthophyll, is one of the dominant carotenoids in diatoms and displays various biochemical properties as antioxidant [3]. Fucoxanthin could work against obesity, diabetes, cancer and angiogenesis and have protective roles among many organisms such as brain, bone and eyes [4]. Commercial applications of fucoxanthin have been explored in last decades [5], and diatoms have been recognized as a preferred source for their higher content of fucoxanthin and capability to grow in controlled bioreactors to avoid outdoor contaminations in comparison with sea weeds. Therefore, rational biotechnological approaches should be developed in order to make fucoxanthin production in diatoms feasible [3, 6].

*Phaeodactylum tricornutum* is a model marine diatom species with a publicly available and annotated whole genome sequence. *P. tricornutum* has three main morphotypes: fusiform, triradiate and oval; a fourth morphotype, cruciform, has also been reported though its occurrence is rare [7]. Diatoms are the key contributor to world biosilicification and one of the major contributors to global carbon fixation [8]. Silicon is one key nutrient for diatoms and is the growth-limiting factor of most diatom species. Cell wall silicification and silicate transport are closely related to cell cycle that the growth rate is partially dependent on the extent of silicification. Cell cycle will stop at G1/S or G2/M transition and the cell morphology will also be disturbed under silicic acid deficiency environment [8]. *P. tricornutum* is an exceptional species, since its frustule is weakly silicified and only one valve of oval cells contains silicon [9]. In most *P. tricornutum* strains, fusiform and triradiate are the major cell morphotypes, whereas oval cells are sparse [10]. Therefore, it is often estimated that silicate has little impact on *P. tricornutum* growth. Different morphotypes help acclimate *P. tricornutum* to different environments: oval cells are more adapted to benthic environment since the oval cells have better sedimentation and surface adhesion; fusiform and triradiate cells are better acclimated to non-sedentary growth condition. Fusiform and triradiate cells transform into to oval cells under stressful situations while oval cells transform into fusiform and triradiate morphotypes under favorable growth conditions [10].

Artificial lighting in microalgal cultivation is usually supplied by fluorescence lamps which emit broad wavelengths that have low photosynthetic efficiency [11]. Recently, light-emitting diodes (LEDs) as novel lighting sources are emerging as superior light sources with the advantages of longevity (approximately 50,000 h or more service life), fast-response, mercury-free and high energy conversion efficiency [11–14]. LEDs emit lights at select wavelengths within a narrow spectrum, which provides suitable photosynthetically active radiation (PAR) for microalgal growth. Absorption of light with 660–680 nm wavelengths usually has the highest quantum efficiency in algae species containing chlorophyll *a* [15, 16]. Red to far-red light (630–750 nm) promotes high growth rates but induces smaller cell size, due to accelerating the cell cycle among some microalgae species [11]. Blue light can impact various metabolic pathways and gene expressions such as the breakdown of endogenous carbohydrate stores [17]. Furthermore, due to the high energy of blue photons, blue light may lead to non-photosynthetic quenching (NPQ) that produces reactive oxygen species (ROS) [17, 18]. Accordingly, algae and plants generate more photoprotective pigments such as xanthophylls to protect cells against ROS.

To promote fucoxanthin accumulation in diatoms, we examined the effects of the silicate concentration and LED illumination on pigments’ accumulation as well as biomass production in *P. tricornutum*. We designed and used synthetic media (named PT-7 and PT-8 medium with 0.3 mM and 3.0 mM silicate, respectively) to cultivate *P. tricornutum* and also applied red light and combined red and blue (50:50) light at different intensities to study light effects on diatoms. Accordingly, this study reported that addition of high silicate and combined red and blue lights mitigated the damaging effect of high light to cells and induce fucoxanthin accumulation. An appropriate setting of the cultivation parameters was established to benefit carotenoid production in diatoms.

## Materials and methods

### Diatom culture and growth conditions

*Phaeodactylum tricornutum* strain (CCAP 1055/1) was acquired from Culture Collection of Algae and Protozoa (CCAP), Scotland, UK. The culture temperature was maintained around 22 ± 2 °C and culture pH was kept around 8.0 ± 0.5. For Erlenmeyer flask culture, the light was continuously provided by the fluorescent lamps with the light intensity at 20 μmol/m^2^/s. The silicate applied in this study is sodium metasilicate (Sigma-Aldrich, St. Louis, USA). A concentration of 0.3 mM silicate is at comparable level or slightly higher than the one in standard f/2 + Si medium as the seawater used to prepare the medium varies slightly in silicate concentration. In this study, both 0.3 mM and 3.0 mM of silicate are sufficient to support high-density culture and not a growth-limiting nutrient based on the elemental composition analysis of *P. tricornutum* biomass. The initial cell densities at all experiments in this study were kept at 0.38 gDCW/L at exponential growth phrase unless otherwise specified. The cells were harvested or examined after 5 days’ continuous cultivation. More detailed description could be seen from our previous publication [3]. The computational metabolic model was applied in this article to estimate the key enzymatic reactions among pigment biosynthesis.

### Growth determination and calculations

The cell number was counted by bright-line hemacytometer (Hausser Scientific, Horsham, UK) and Leica DMIRB microscopy in triplicates and cell concentration (cells/mL) was utilized to calculate growth rate. The biomass dry weight or dry cell weight (DCW) was primarily measured by collecting cells on a cellulose membrane with 0.45 μm pore size. The cellulose membrane was then washed twice with deionized water to remove salts and dried at 60 °C overnight before weighing. The optical density at 625 nm wavelength (OD625) was also applied to estimate the biomass production. The detailed correlation between biomass production and OD625 was demonstrated in previous publication [6]. The unit of the pigment yields that we apply in this study was the dry biomass percentage (mg/g DCW). The biomass yield in Table 1 represented the biomass yield on light energy with a unit of gram per mol photons.

**Table 1:** Effect of different incident photon fluxes on the growth of *P. tricornutum* under red LED illumination

Photon flux (μmol/m^2^/s)	Biomass productivity (gDCW/L/day)		Biomass yield (gDCW/mol)	
	PT-7 medium	PT-8 medium	PT-7 medium	PT-8 medium
85	0.19 ± 0.004	0.19 ± 0.01	0.26 ± 0.01	0.25 ± 0.01
128	0.21 ± 0.01	0.23 ± 0.01	0.19 ± 0.01	0.21 ± 0.01
170	0.24 ± 0.01	0.31 ± 0.01	0.16 ± 0.01	0.21 ± 0.005
204	0.32 ± 0.02	0.36 ± 0.01	0.18 ± 0.01	0.21 ± 0.01
255	0.34 ± 0.02	0.37 ± 0.03	0.15 ± 0.01	0.17 ± 0.01

*P. tricornutum* was cultivated using PT-7 and PT-8 medium, respectively for batch culture of 5 days with red LED light illumination. The results presented are average values from three independent experiments (mean ± standard error of mean (SE))

### LED light setup and photobioreactors

Since red light has longer wavelength (it produces more photons and excites more pairs of chlorophylls than blue light under same luminous energy), the red light usually has the highest quantum efficiency. Nevertheless, excess red light causes photo damaging effect to algal cells. Additional blue light could promote photoprotective effect, accordingly, we applied the pure red light and red + blue light (50:50) as illuminant source in this study. The artificial light supply was setup with red LED light (Part number: SSL-LX5093SRC, LUMEX, Taiwan) and blue LED light (Part number: VAOL-5LSBY2, LUMEX, Taiwan) based on (Al, Ga) InPsystem [12–14]. The average photon flux was provided with a frequency of 10 kHz and the light intensity was manipulated by controlling the duty cycle. The central wavelengths for red and blue light were at 660 nm and 470 nm respectively, with a 20 nm bandwidth for both output spectra. The composition of PT-7 and PT-8 media was shown in Additional file 1: Table S1.

### LC–MS detection and analysis of pigments

The detailed procedure for LC–MS detection was described previously [13, 14]. Briefly, 0.5 mL aliquot of cell culture was centrifuged 10 min at 2000×*g.* The cell pellet was collected and resuspended with ethanol and hexane mixture (2:1 v/v with 3.0 mL in total). De-ionized water at 2.0 mL and 4.0 mL of hexane was then added into the mixture. The samples were then vigorously vortexed and re-centrifuged for 5 min at 2000×*g*. Hexane layer was transferred to miVac Quattro (Genevac, England) and evaporated at room temperature, then re-dissolved with methyl tertiary butyl ether (MTBE): acetonitrile (ACN) (1:1 v/v). The liquid was analyzed by ultra-high performance liquid chromatography, coupled with UV and mass spectrometer (UPLC-UV–MS). UPLC separation was performed on ACQUITY UPLC (Waters, Milford, USA) and an HSS T3 1.8 μm column (2.1 × 150 mm; Waters, UK) was applied for reversed phase chromatography.

### Morphological analysis and transmission electron microscopy

The cell number of each morphotype was counted by bright-line hemacytometer (Hausser Scientific, Horsham, UK) with Leica DMIRB microscopy at 0 day, 6 day and 12 day of PT-7 and PT-8 medium cultivation. And for the transmission electron microscopy process: Diatom samples were transferred to eppendorf tubes and fixed in 2.5% glutaraldehyde for 20 min [19]. Then the cells were centrifuged for 2 min in 6688×*g* and the supernatant were removed. Cells were washed in phosphate buffer twice for 2 min and then centrifuged again for 2 min in 6688×*g*. Cells were fixed in 2% osmium tetroxide for 30 min, then were rinsed twice with phosphate buffer for 3 min. Cells were dehydrated in ethanol in series: incubated in 25% ethanol for 2 min, 50% ethanol for 2 min, 70% ethanol for 2 min, followed by 80% ethanol for 2 min, 90% ethanol for 2 min, 96% ethanol first for 2 min, then incubated with 96% ethanol twice for 7 min. The cells were then incubated with resin and 96% ethanol mixture (1:1, volume) for 1 h. Then the mixture was replaced with pure resin and fixed for 1 h in room temperature. The tubes were transferred into 70 °C oven overnight. T blocks were cut off from the tubes to Ultramicrotome Leica EM UC7 for further processing. Sections on grids were imaged using a JEM-1400PLUS PL Transmission Electron Microscope (JEOL, Japan) at various magnifications.

### Pathway and model analysis

The carotenoids, lipids and chlorophyll biosynthesis pathways are cited from the KEGG database (Kyoto Encyclopedia of Genes and Genomes, Japan). The *i*LB1025 genome-scale computational model was applied in this study to predict the linearly correlated reactions and enzymes that associated with fucoxanthin accumulation. Detailed method was described in our previous publication [6].

## Results

### Morphological analysis of *P. tricornutum* cells under PT-7 and PT-8 medium

**Fig. 1:**
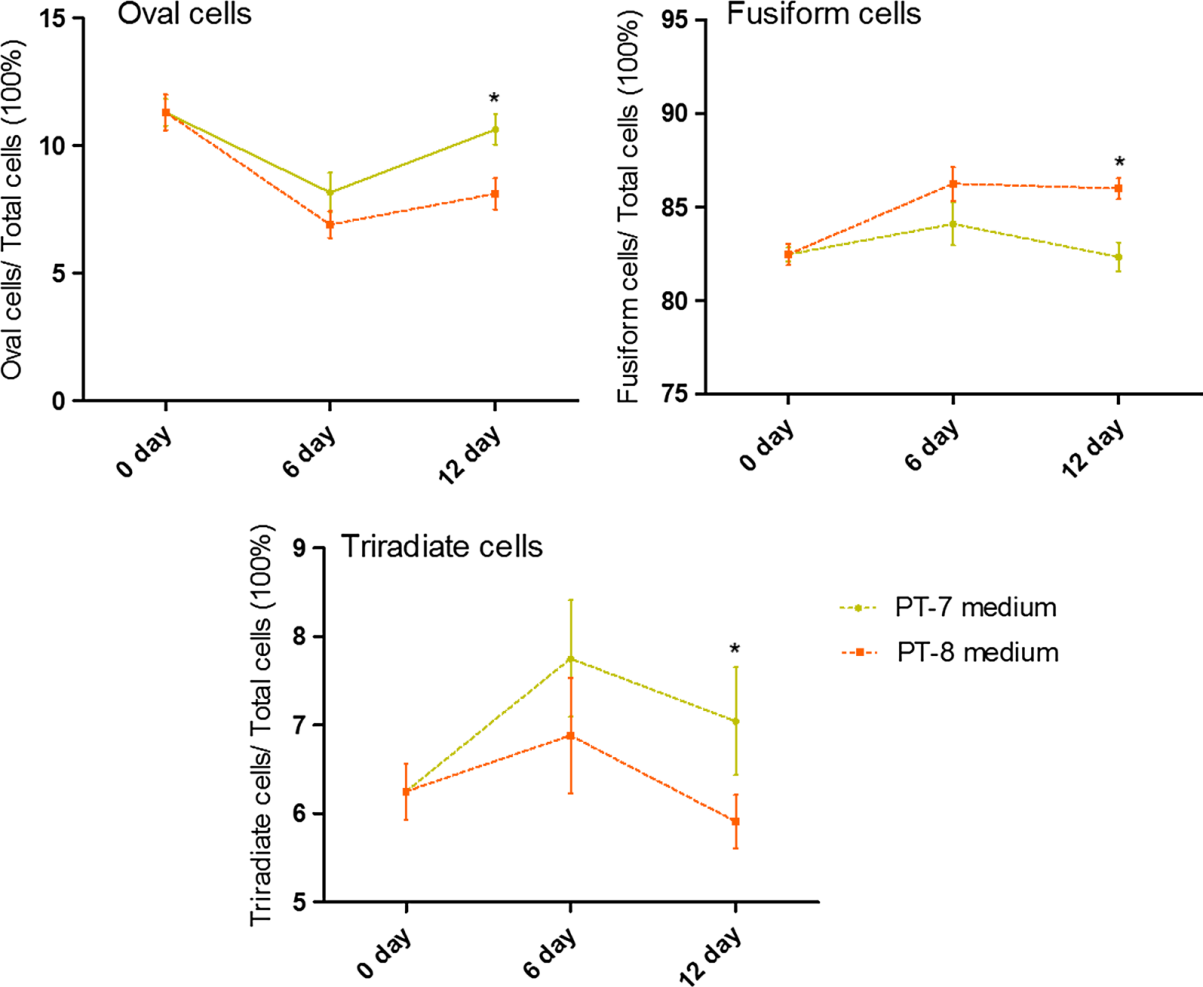
Analysis of cell morphology in batch culture. Cells were cultivated in PT-7 and PT-8 medium respectively, in batch culture over 12 days under fluorescent lamp irradiation with a light intensity of 20 μmol/m^2^/s. Cells were counted by hemocytometer and data were averaged from 3 independent experiments. Student’s *t*-test was applied to analyze the group difference, asterisks represent statistically significant differences between the groups (P < 0.05)

There are three major morphotypes in *P. tricornutum*: Fusiform, triradiate, and oval forms. These three morphotypes can interconvert under certain environmental conditions [20]. In this study, we cultivated *P. tricornutum* with PT-7 and PT-8 medium (as shown in Additional file 1: Table S1) in batch culture over 12 days in Erlenmeyer flask (Fig. 1). For oval cells (11.3% in the starting population), the proportion was changed to 8.17% and 10.63% at day 6 and day 12, respectively, in PT-7 medium, while the proportion decreased to 6.9% and 8.11% at day 6 and day 12, respectively, in PT-8 medium. The proportion of fusiform cells remained unchanged (from 82.45 to 82.32%) over 12 days culture in PT-7 medium while fusiform cells increased to 85.98% at day 12 in PT-8 medium. Triradiate cell form accounted for 6.25% of total population at the beginning and it was 7.04% in PT-7 medium and 5.91% in PT-8 cultivation, respectively, at the end of 12 days cultivation.

**Fig. 2:**
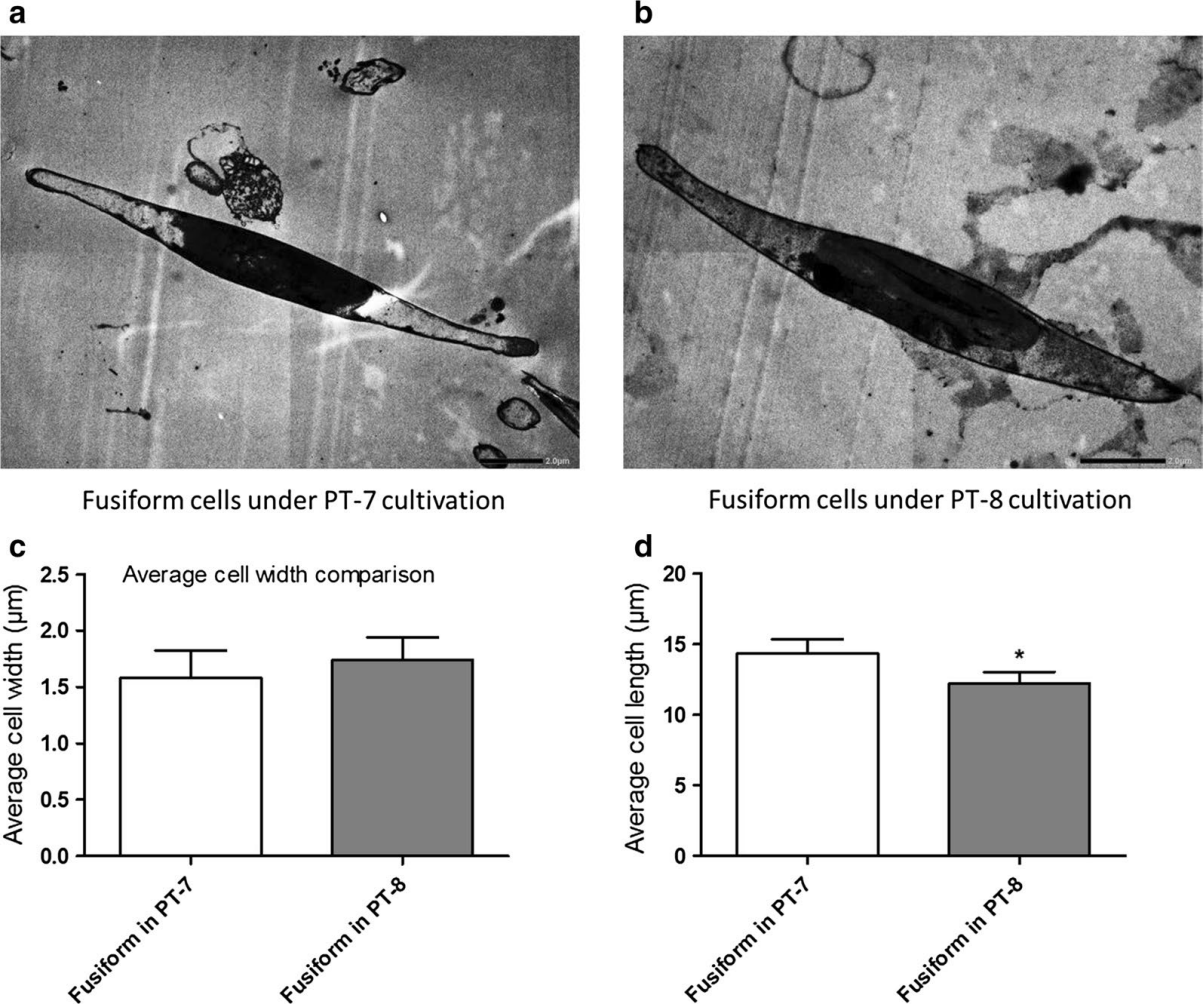
Fusiform cell size comparison. The *P. tricornutum* cells were collected at 12 days of PT-7 or PT-8 medium cultivation and then observed by transmission electron microscopy (TEM). The fusiform cell width and length were averaged from 100 fusiform cells for each sample. The *t*-test was applied to compare the group differences and asterisk represented the significant difference between two groups (P < 0.05)

According to our observations with TEM (Fig. 2), the majority of fusiform cells in PT-7 medium were slightly longer than cells in PT-8 medium. The average fusiform cell length in PT-7 medium (14.33 ± 1.43 µm) is longer than cells in PT-8 medium (12.20 ± 0.67 µm) (P = 0.0424) while the average fusiform cell width in PT-7 medium is 1.58 ± 0.25 µm, which is similar as cells in in PT-8 medium (1.74 ± 0.23 µm) (P = 0.2490). This phenomenon was also observed in other diatom species: The cell volume of *Chaetoceros debilis* increases up to three times under silicate limited conditions [21]. The mechanism for the elongation is still not clear, it is estimated that the elongation process is part of an adaptation response that the cell might employ to acquire more silicate or other limited nutrients [21].

### Increasing metasilicate concentration in the medium from 0.3 mM (PT-7) to 3.0 mM (PT-8) enhanced growth under higher light intensities

*Phaeodactylum tricornutum* was cultivated under 100% red LEDs with five different photon fluxes in both PT-7 and PT-8 medium, and the average growth rate at each photon flux was measured (Table 1).

*Phaeodactylum tricornutum* exhibited an increased growth rate with enhanced light intensity at both PT-7 and PT-8 medium. *P. tricornutum* had an identical growth rate of 0.19 gDCW(dry cell weight)/L/day at both media under 85 μmol/m^2^/s light illumination, and the growth rate was higher under PT-8 medium cultivation than PT-7 medium while the photon flux intensity exceeded 128 μmol/m^2^/s. The growth rate for PT-7 and PT-8 medium cultivation was 0.24 ± 0.01 gDCW/L/day and 0.31 ± 0.01 gDCW/L/day, respectively, at 170 μmol/m^2^/s red light illumination. The biomass yield of PT-7 medium cultivation decreased gradually from 0.26 ± 0.01 to 0.15 ± 0.01 gDCW/mol when the light illumination increased from 85 to 255 μmol/m^2^/s. The biomass yield of PT-8 medium cultivation reduced from 0.25 ± 0.01 to 0.17 ± 0.01 gDCW/mol when the light intensity increased from 85 to 255 μmol/m^2^/s.

### Effects of PT-7 and PT-8 medium on the pigments accumulation of *P. tricornutum* under different light intensities

**Fig. 3:**
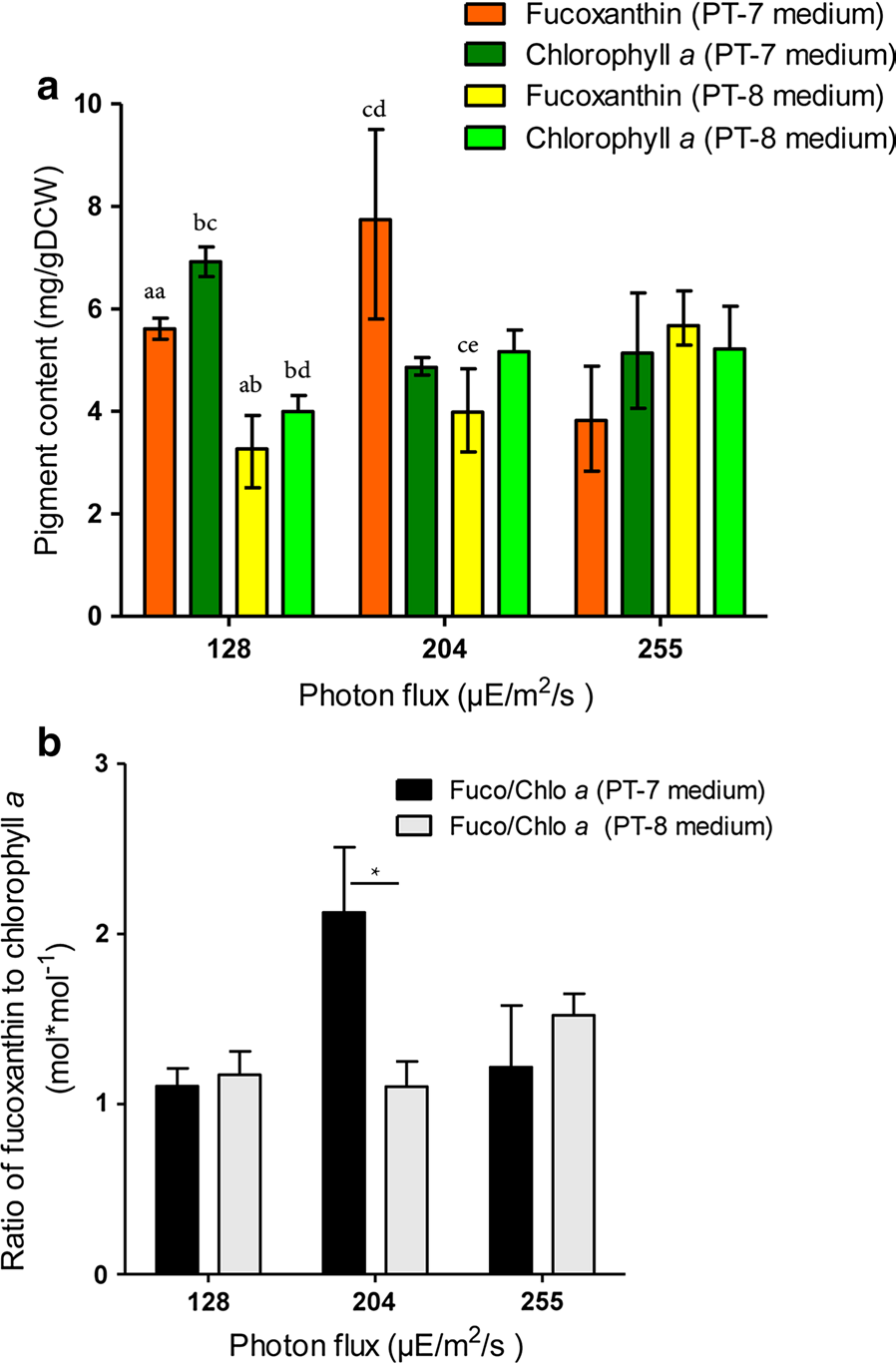
Effects of different incident photon fluxes on the accumulation of fucoxanthin and chlorophyll *a* in *P. tricornutum* under red LED illumination. **a** Fucoxanthin and chlorophyll *a* content in PT-7 and PT-8 medium under different incident photon fluxes; **b** ratio of fucoxanthin to chlorophyll *a* under different incident photon fluxes. The results are average values from either two or three independent experiments. Error bars indicate SE. Asterisk (*) represents statistically significant difference of the ratio between the two groups. The symbols aa and ab represents statistically significant difference of pigment content between the two groups. The symbols bc and bd represents statistically significant difference of pigment content between the two groups. The symbols cd and ce represents statistically significant difference of pigment content between the two groups

*Phaeodactylum tricornutum* was cultivated in both PT-7 and PT-8 medium under red light illumination at 128, 204 and 255 μmol/m^2^/s photon flux. Pigments were extracted and LC–MS was utilized to identify and quantify important pigments. As shown in Fig. 3, 0.3 mM silicate and 3.0 mM silicate had different impacts on fucoxanthin and chlorophyll *a* production at different photon fluxes. *P. tricornutum* accumulated more fucoxanthin and chlorophyll *a* at 0.3 mM silicate medium than 3.0 mM silicate under 128 μmol/m^2^/s illumination. Nonetheless, the fucoxanthin/chlorophyll *a* ratio remained stable under PT-7 and PT-8 medium cultivation. Fucoxanthin content was the highest among all stimulation groups under 204 μmol/m^2^/s irradiation with 0.3 mM silicate culture but dropped to 4.3 mg/gDCW under 3.0 mM silicate culture. The chlorophyll *a* content was almost identical under 0.3 mM and 3.0 mM silicate medium. The ratio of fucoxanthin/chlorophyll *a* under 0.3 mM silicate cultivation was approximately twice of the ratio under 3.0 mM silicate cultivation. For 255 μmol/m^2^/s red light irradiation, both fucoxanthin and chlorophyll *a* contents were reduced in 0.3 mM silicate culture in comparison with that in 3.0 mM silicate culture.

*Phaeodactylum tricornutum* accumulated approximately 1.5 times beta-carotene content at 204 μmol/m^2^/s than at 128 μmol/m^2^/s under both PT-7 medium and PT-8 medium (Table 2). *P. tricornutum* accumulated approximate 2.1 times and 3.8 times more beta-carotene under 255 μmol/m^2^/s illumination than under 128 μmol/m^2^/s at PT-7 and PT-8 medium, respectively, indicating high silicate promoted higher beta-carotene production under high light irradiation. For diadinoxanthin, *P. tricornutum* had around 25% higher production under both 204 μmol/m^2^/s and 255 μmol/m^2^/s illumination compared with 128 μmol/m^2^/s illumination in PT-7 medium while higher light irradiation resulted in less accumulation of diadinoxanthin in PT-8 medium. Violaxanthin accumulation exhibited similar trend as diadinoxanthin in PT-7 and PT-8 medium while beta-cryptoxanthin had an opposite profile under higher light conditions: *P. tricornutum* accumulated less beta-cryptoxanthin in PT-7 medium under higher light conditions than 128 μmol/m^2^/s irradiation but had higher beta-cryptoxanthin production than the content under 128 μmol/m^2^/s irradiation in PT-8 medium.

**Table 2:** Effect of two different levels of silicate in media (PT-7 and PT-8) on the accumulation of carotenoids in *P. tricornutum* under elevated light intensities

Photon flux (μmol/m^2^/s)	128 (%)	204 (%)	255 (%)
β-Carotene			
PT-7	100	257.6 ± 0.4*	312.7 ± 23.4*
PT-8	100	239.6 ± 0.5*	475.3 ± 19.2*
Diadinoxanthin			
PT-7	100	123.4 ± 0.4*	125.1 ± 4.1*
PT-8	100	75.3 ± 4.5*	72.8 ± 6.6*
Violaxanthin			
PT-7	100	119.7 ± 0.6*	151.3 ± 2.2*
PT-8	100	96.5 ± 9.1^NS^	75.9 ± 7.3*
β-Cryptoxanthin			
PT-7	100	90.5 ± 11.2^NS^	90.6 ± 7.9^NS^
PT-8	100	144.6 ± 23.4*	157.5 ± 2.9*
Phoenicoxanthin			
PT-7	100	95.1 ± 2.9^NS^	109.4 ± 2.0^NS^
PT-8	100	55.0 ± 1.5*	57.6 ± 6.2*

Data were averaged from either two or three independent experiments (mean ± SE). Contents of carotenoid species in cells growing on PT-7 and PT-8 medium under a pure (or 100%) red light with an intensity of 128 μmol/m^2^/s were set as references (100%), respectively

Asterisk (*) indicates statistically significant difference between its low light condition and higher (204 or 255 μmol/m^2^/s) light condition

NS represents no statistically significant difference between its low light condition and higher (204 or 255 μmol/m^2^/s) light condition

### Effects of red light and combined red and blue (50:50) light on the pigments accumulation in *P. tricornutum*

**Fig. 4:**
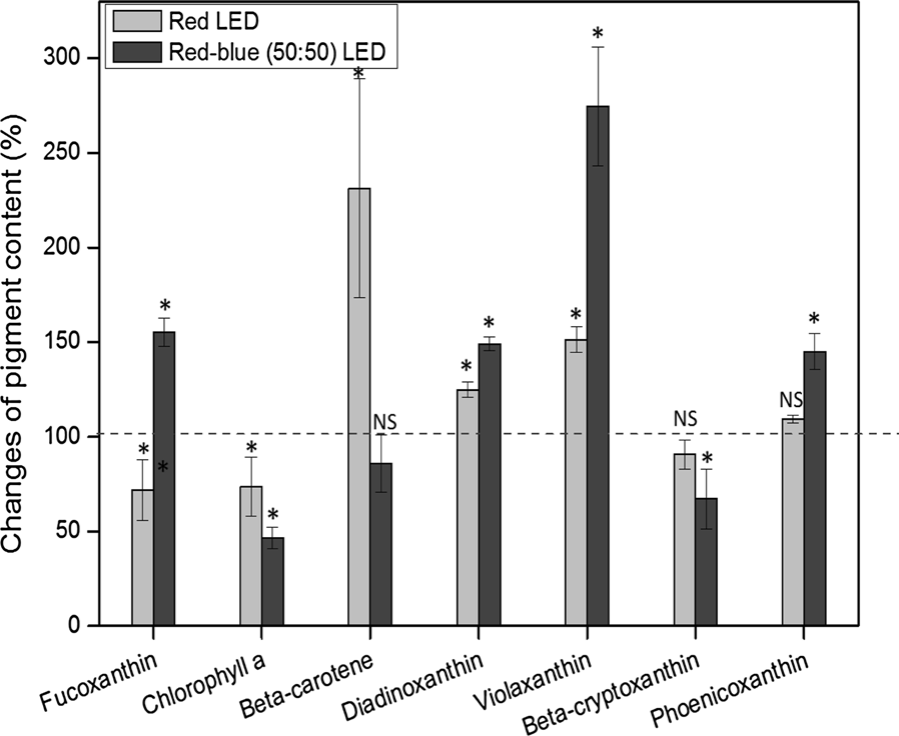
Changes of fucoxanthin and other major pigments in *P. tricornutum* under LED illumination after doubling light intensities. Because light energy differs in different wavelengths, the pure red light with a light intensity of 128 μmol/m^2^/s is approximately equal to the combined red and blue (50:50) light with a light intensity of 102 μmol/m^2^/s in terms of light energy. To setup same supplied light energy as a baseline, different light intensities were used for two different light qualities. Red LED conditions: light intensity was increased to 255 from 128 μmol/m^2^/s. Red-blue (50:50) LED conditions: light intensity was increased to 204 from 102 μmol/m^2^/s. Due to the The pigment contents in cells under illumination of lower light intensities were set as references (100%), respectively. The dashed line indicates the baseline of 100%. The results presented are average values from three independent experiments. Error bars indicate SE. Asterisk (*) represents statistically significant difference of the pigment content between its lower light condition and doubled light condition. NS represents no statistically significant difference between its low light condition and high (doubled) light condition

In order to check different light qualities on *P. tricornutum* pigments accumulation, we doubled both the red light and combined red and blue (50:50) light intensity and compared the achieved pigment contents with the ones under PT-7 medium (Fig. 4). Fucoxanthin, chlorophyll *a* and beta-cryptoxanthin content dropped 27.5%, 28.3% and 8.6% under 255 μmol/m^2^/s red light illumination compared with the contents under 128 μmol/m^2^/s red LEDs light irradiation. On the contrary, beta-carotene, diadinoxanthin, violaxanthin and phoenicoxanthin content enhanced by 162.3%, 21.5%, 51.3% and 9.8%, respectively. Chlorophyll *a*, beta-carotene and beta-cryptoxanthin content reduced by 53.2%, 17.2% and 29.3% when the combined red and blue (50:50) light intensity doubling to 204 from 102 μmol/m^2^/s. The fucoxanthin, diadinoxanthin, violaxanthin and phoenicoxanthin content increased by 53.8%, 47.6%, 173.2%, and 35.1%, respectively, compared with the contents under 102 μmol/m^2^/s light illumination.

### Effects of red and blue (50:50) LED illumination on the growth and fucoxanthin content of *P. tricornutum*

**Fig. 5:**
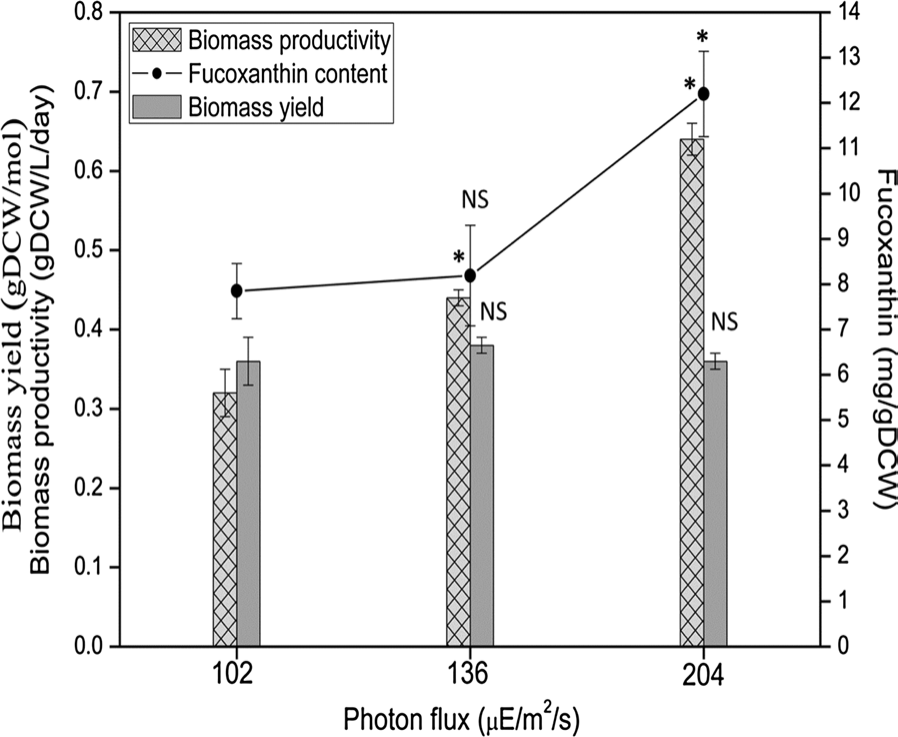
Effects of different incident photon fluxes on the growth and fucoxanthin content in *P. tricornutum* under combined red and blue (50:50) LED illumination. The results presented are average values from three independent experiments. Error bars indicate SE. Asterisk (*) represents statistically significant difference of growth data or fucoxanthin content at higher light conditions (136 or 204 μmol/m^2^/s) in comparison with the light condition at 102 μmol/m^2^/s. NS represents no statistically significant difference

In order to test the effects of combined red and blue (50:50) LED illumination on diatom growth and fucoxanthin accumulation, different light intensities, i.e. 102, 136 and 204 μmol/m^2^/s, were applied individually. The intensity of combined red and blue (50:50) LED light had positive correlations with both the biomass productivity and fucoxanthin content: the biomass productivity enhanced from 0.32 to 0.63 gDCW/L/day and fucoxanthin content increased from 7.5 to 12.2 mg/gDCW as the light intensity increased from 102 to 204 μmol/m^2^/s (Fig. 5). Besides, the biomass yield at 102 μmol/m^2^/s was close to the yield at 136 and 204 μmol/m^2^/s. The red LED light had the best economic efficiency of energy to biomass compared with other colors but pure red light illumination can cause photo-oxidative damage [22]. Considering that the biomass yield dropped gradually from 85 to 255 μmol/m^2^/s under 100% red light irradiation (Table 1), combined red and blue (50:50) LED light may be a good irradiation combination to enhance the biomass production as well as fucoxanthin production for industrial applications.

## Discussion

The effects of silicate on the morphotype transformation of *P. tricornutum* under certain circumstances have been studied [9]. In general, the oval cells are better adapted to unfavorable environments and they may change into fusiform and triradiate form under suitable growth conditions. There are less oval cells in PT-8 medium than PT-7 medium (as shown in Fig. 1) despite oval cells being the only cell type in *P. tricornutum* that requires silicon for mitosis [23]. Because oval cells usually emerge during *P. tricornutum* adaptation under stress conditions, it is speculated that PT-8 medium is more favorable to growth than PT-7 medium. Based on this phenomenon (Fig. 1) and the results (Table 1) that *P. tricornutum* had higher growth rate at 3.0 mM silicate medium under high light conditions, it is implied that *P. tricornutum* is more inclined to grow at 3.0 mM silicate medium (PT-8) than 0.3 mM silicate medium (PT-7).

Previous studies indicated that *P. tricornutum* requires little silicon for normal growth since its cell walls are not heavily silicified in the absence of stressors [24]. Nevertheless, most of these studies were conducted at low light intensity [25–27]. In this study, silicon increased the biomass productivity under red LED light irradiation at modest to high light intensity (Table 1). Fucoxanthin, chlorophyll *a* and fucoxanthin-chlorophyll *a*/*c* binding proteins (FCPs) form the light harvesting antenna in diatoms, which is different from light harvesting complexes (LHC) in high plants [28]. Research showed that the concentration of fucoxanthin and chlorophyll *a* dropped 8.4% and 16.8% after 2 days of silicon starvation in *P. tricornutum* and FCPs were also down regulated [10]. A genome-wide transcriptome microarray results demonstrated that 13 genes were up-regulated under Si-starved medium while 210 genes were up-regulated under complete medium [25]. The gene coding FCP (p54065) was down-regulated during Si-limitation, which is also consistent with proteomic data [10, 25]. The protein (B7FP19), which was predicted to modulate chlorophyll *a* synthesis, was also down-regulated under Si-limitation [10].

**Fig. 6:**
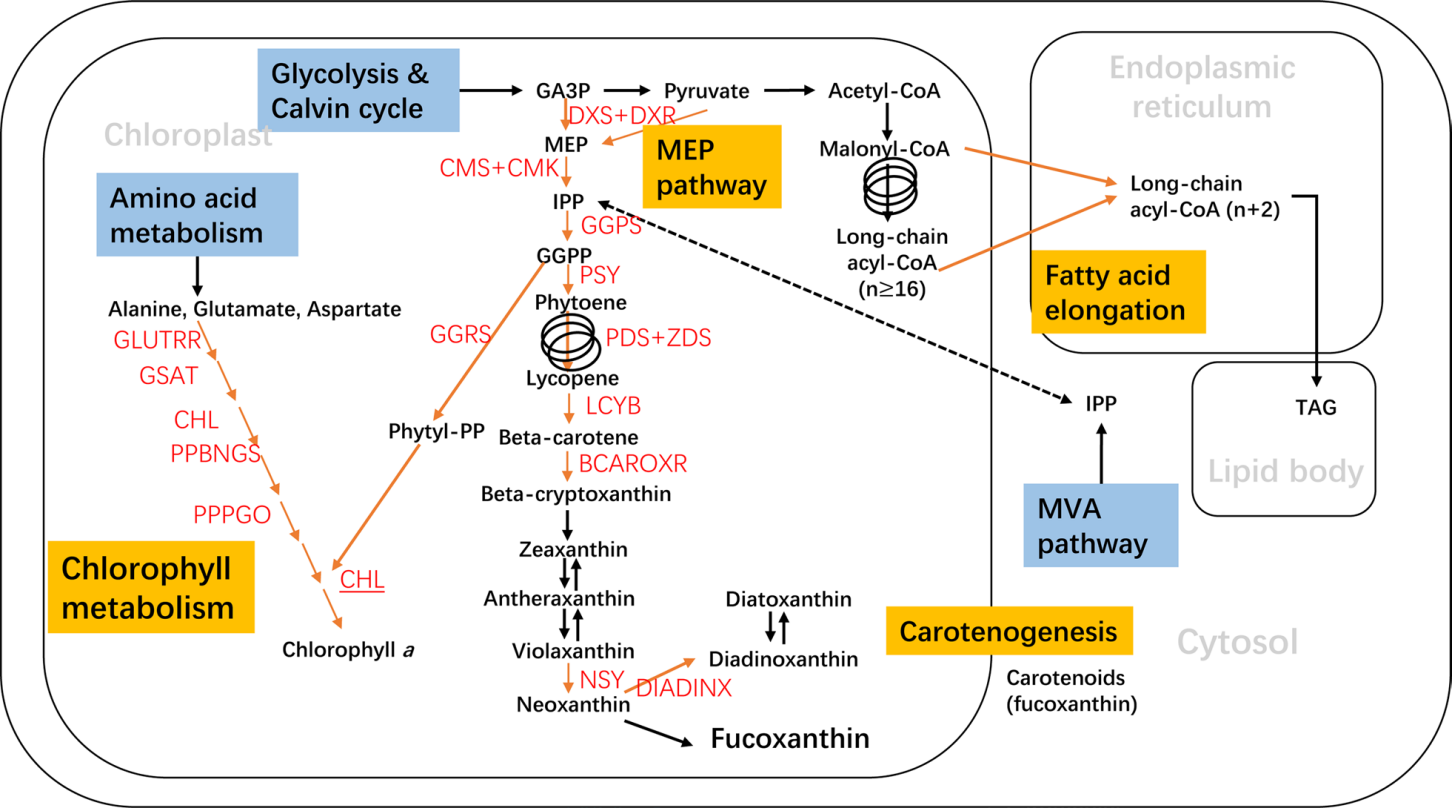
Interconnected metabolic pathways of carotenoids, chlorophylls and lipid synthesis. The reactions and enzymes that were predicted by the iLB1025 computational model to have the linear correlation with fucoxanthin accumulation were labeled as red color. GA3P, glyceraldehyde 3-phosphate; MEP, methylerythritol 4-phosphate; IPP, isopentenyl pyrophosphate; GGPP, geranylgeranyl pyrophosphate; MVA, mevalonate; TAG, triacylglycerol; DXS, 1-deoxy-D-xylulose 5-phosphate synthase; DXR, 1-deoxy-D-erythritol 2, 4-cyclodiphosphate synthase; PSY, phytoene synthase; PDS, phytoene desaturase; ZDS, zeta-carotene desaturase; LCYB, lycopene cyclase; BCAROXR, beta-carotene, NADH; oxygen 3-oxidoreductase; NSY,neoxanthin synthase; DIADINX, Diadinoxanthin synthase; GLUTRR, glutamyl-tRNA reductase; GSAT, glutamate-1-semialdehyde aminotransferase; CHL, chlorophyll synthase [6]

Carotenoids as one of the major antioxidants in diatoms have interconnected metabolic pathways with chlorophyll and lipid metabolism (as shown schematically in Fig. 6). Both fucoxanthin and chlorophyll *a* were down-regulated in *P. tricornutum* when exposed to high light illumination, which is consistent with previous research [29–31]. The computational models based on genome and biochemical data are emerging to be a novel approach to comprehend and estimate the correlations among the comprehensive enzymatic reactions and metabolites [32]. MEP (2-*C*-methyl-d-erythritol 4-phosphate cytidylyltransferase) is the common precursor of both chlorophylls and carotenoids and it was predicted by the iLB1025 model that MEP had linear association with fucoxanthin production. MEP is converted from DXP (1-Deoxy-D-xylulose 5-phosphate) via catalysis of DXS (DXP synthase) (Fig. 6). The gene coding for DXS was down-regulated in *P. tricornutum* while acclimating high light. Therefore, this could partially explain the decline for both chlorophyll *a* and fucoxanthin. Besides, under high light irradiation, genes encoding other enzymes in chlorophyll *a* biosynthesis were also significantly reduced on the transcriptional level [29].

In Table 2, phoenicoxanthin content dramatically decreased and beta-cryptoxanthin increased in 3.0 mM silicate medium while both phoenicoxanthin and beta-cryptoxanthin kept relatively stable in 0.3 mM silicate medium under high light conditions. Beta-carotene is the precursor of both phoenicoxanthin and beta-cryptoxanthin: phoenicoxanthin is catalyzed by beta-carotene hydroxylase (*crtZ*) and ketolase (*bkt*) from beta-carotene while beta-cryptoxanthin is catalyzed only by beta-carotene hydroxylase (*crtZ*) [33]. The enzyme *crtZ* is known to be highly enhanced in transcript levels in many other species under high light or other stress conditions [33–37]. Consequently, it is speculated that high silicate enhanced *crtZ* transcript level and/or activated *crtZ* catalytic capacity in *P. tricornutum* under high light conditions; on the contrary, *bkt* was reduced or de-activated by high silicate under high light that resulted in a reduction of phoenicoxanthin level. Although it was reported that silicon had little impact on the growth of *P. tricornutum* under normal conditions, 223 genes including these involved in carbohydrate metabolism and urea acquisition were found differentially modulated with the change of silicate concentration [25], indicating that silicon plays an important role in regulating various physiological activities.

Non-photochemical quenching (NPQ) is the primary short-term protection mechanism that dissipates harmful excess energy into heat. The up-regulation of these pigments was estimated to provide extra anti-oxidative protection [33]. NPQ is usually regulated by de-epoxidation reactions of xanthophylls [33, 34]. The main de-epoxidation reaction in diatoms is triggered by diadinoxanthin cycle, which converts diadinoxanthin into diatoxanthin under high light irradiations. Table 2 shows an increase in diadinoxanthin under elevated light intensity in PT-7 medium but not in PT-8 medium. Under high silicate and high light condition, the increased fucoxanthin and chlorophyll *a* production but decreased diadinoxanthin might be due to the activated biosynthesis of stress tolerance proteins. Polyubiquitin and annexin are stress tolerance proteins that modulate stress signaling and are essential for the resistance to external stresses [35, 36]. Silicon can increase the production of polyubiquitin and annexin in *P. tricornutum* [10]. With more stress-tolerance proteins produced, the cell stress-tolerance capacity may be enhanced, protecting fucoxanthin and chlorophyll *a* levels in *P. tricornutum* from being down-regulated by high-light irradiation.

It has been known for many decades that irradiating plants with blue and red lights may fulfill particular photosynthetic needs [37]. Blue light is known to induce enzymatic activation and regulate gene expressions that relate to photoprotection responses [17] and can partially reverse the damaging effect of red light [37]. In this study, fucoxanthin content was up-regulated by enhanced red and blue (50:50) LED. Considering that other xanthophylls like diadinoxanthin, violaxanthin, and phoenicoxanthin that share biosynthetic pathways were also up-regulated, it is speculated that the blue light activated enzymes that increase xanthophyll synthesis. Xanthophyll cycle involves an enzymatic reaction to remove the epoxy groups from xanthophylls to create de-epoxidised xanthophylls. The enzymatic reactions help to dissipate excess energy from photosynthetic antenna by non-photochemical quenching (NPQ) [38]. Most xanthophylls had higher induction with combined red and blue (50:50) LED irradiation than red light alone at a similar light intensity (Fig. 4), which indicated that blue light play an important role in promoting photo-protection. Due to lack of equipment, we did not measure the Pulse-amplitude modulation (PAM) of fluorescence. It would be extremely helpful to apply PAM fluorometry to explore the pigments’ production under NPQ. White light which is the most common illuminant is widely distributed in almost every inhabited place. It was formed with light at different wavelengths including red and blue light [31]. According to this study, it is speculated that high white light combined with high silicate cultivation could also boost fucoxanthin production that would further contribute to the universality and practical convenience of the algal cell factory platform.

## Conclusions

Supplementation with silicate at 3.0 mM reversed the down-regulation of fucoxanthin and chlorophyll *a* from the photon damage under high red LED illumination. Both fucoxanthin content and biomass productivity increased with increased red and blue (50:50) light intensity. The findings in this study could deepen our understanding of diatom metabolism and promote the yields of both the biomass and fucoxanthin towards industrial applications.

## Supplementary information

**Additional file 1.** Additional figures and table.

## Data Availability

All data needed to evaluate the conclusions are present in the paper and/or Additional file 1.

## Abbreviations

*P. tricornutum*: *Phaeodactylum tricornutum*
LEDs: light-emitting diodes
PAR: photosynthetically active radiation
NPQ: non-photosynthetic quenching
ROS: reactive oxygen species
OD: optical density
MTBE: methyl tertiary butyl ether
ACN: acetonitrile
UPLC: ultra-high performance liquid chromatography
TEM: transmission electron microscopy
FCPs: fucoxanthin-chlorophyll *a*/*c* binding proteins
LHC: light harvesting complexes
GA3P: glyceraldehyde 3-phosphate
MEP: methylerythritol 4-phosphate
IPP: isopentenyl pyrophosphate
GGPP: geranylgeranyl pyrophosphate
MVA: mevalonate
TAG: triacylglycerol
DXS: 1-deoxy-d-xylulose 5-phosphate synthase
DXR: 1-deoxy-d-erythritol 2,4-cyclodiphosphate synthase
PSY: phytoene synthase
PDS: phytoene desaturase
ZDS: zeta-carotene desaturase
LCYB: lycopene cyclase
BCAROXR: beta-carotene, NADH, oxygen 3-oxidoreductase
NSY: neoxanthin synthase
DIADINX: diadinoxanthin synthase
GLUTRR: glutamyl-tRNA reductase
GSAT: glutamate-1-semialdehyde aminotransferase
CHL: chlorophyll synthase
