# Supplementary material for: Combined artificial high-silicate medium and LED illumination promote carotenoid accumulation in the marine diatom Phaeodactylum tricornutum
Source: Microb Cell Fact. 2019 Dec 2;18:209. doi: 10.1186/s12934-019-1263-1 (PMC6889666; doi:10.1186/s12934-019-1263-1)
Supplement: Supplementary file 1 — Additional file 1. Additional figures and table. [file 12934_2019_1263_MOESM1_ESM.docx]

*Microbial Cell Factories*

Combined artificial high-silicate medium and LED illumination promote carotenoid accumulation in the marine diatom *Phaeodactylum tricornutum*

Zhiqian Yi^1,2,5^, Yixi Su^1^, Paulina Cherek^5^, David R. Nelson^3^, Jianping Lin^4^, Ottar Rolfsson^1^, Hua Wu^2^, Kourosh Salehi-Ashtiani^3^, Sigurdur Brynjolfsson^1^, Weiqi Fu^1,3,*^

^1^ Center for Systems Biology and Faculty of Industrial Engineering, Mechanical Engineering and Computer Science, School of Engineering and Natural Sciences, University of Iceland, Reykjavík 101, Iceland;

^2^ Department of Orthopaedics, Tongji Hospital, Tongji Medical College, Huazhong University of Science and Technology, Wuhan, China 430030;

^3^Center for Genomics and Systems Biology, and Division of Science and Math, New York University Abu Dhabi, Abu Dhabi 129188,

^4^Key Laboratory of Biomass Chemical Engineering of the Ministry of Education, College of Chemical and biological Engineering, Zhejiang University, Hangzhou, 310027, China;

^5^Biomedical Center and Department of Anatomy, Faulty of Medicine, University of Iceland, Reykjavík 101, Iceland;

*****Author to whom correspondence should be addressed: WF, [weiqi@hi.is](mailto:weiqi@hi.is); Tel: +971-563052428

**Figure S1**

**Fig. S1** Growth of *P. tricornutum* on silicate free PT-6 medium under red LED and combined red and blue (50:50) LED illumination. The light intensities were 128 μE/m^2^/s for red light and 102 μE/m^2^/s for combined red and blue lights, respectively. The results presented are average values from three independent experiments. Error bars indicate SD.

**Figure S2**

**Fig. S2** Effect of different incident photon fluxes on the chlorophyll *a* and beta-carotene content in *P. tricornutum* under combined red and blue (50:50) LED illumination. The results presented are average values from three independent experiments. Error bars indicate SD.

**Table S1** Nutrient composition of media PT-8 and PT-7

| Compound | PT-8 (mg/L) | PT-7 (mg/L) |
| --- | --- | --- |
| NaCl | 29250 | 29250 |
| MgSO_4_ ·7H_2_O | 1232 | 1232 |
| H_3_BO_3_ | 124 | 124 |
| KNO_3_ | 3150 | 3150 |
| Na_2_SiO_3_ | 366 | 36.6 |
| Na_2_HPO_4_ | -- | 568 |
| NaH_2_PO_4_ ·H_2_O | 552 | -- |
| NaFeEDTA | 10.0 | 10.0 |
| Cyanocobalamin (Vitamin B_12_) | 0.0135 | 0.0135 |
| Thiamine HCl (Vitamin B_1_) | 0.5 | 0.5 |
| Biotin | 0.001 | 0.001 |
| CuSO_4_ · 5H_2_O | 0.75 | 0.75 |
| ZnSO_4_ · 7H_2_O | 4.6 | 4.6 |
| CoCl_2_ · 6H_2_O | 0.24 | 0.24 |
| MnCl_2_ · 4H_2_O | 0.69 | 0.69 |
| Na_2_MoO_4_ · 2H_2_O | 0.36 | 0.36 |
| CaCl_2_ ·2H_2_O | 13.0 | 13.0 |
